# Sudden Suppression

**Published:** 1895-04

**Authors:** 


Sudden suppression of an excessive salivation occurring during pregnancy, Prof. Parvin says, may be followed by serious consequences.

